# The study on interacting factors and functions of *GASA6* in *Jatropha curcas* L.

**DOI:** 10.1186/s12870-023-04067-4

**Published:** 2023-02-18

**Authors:** Xue Li, Ming-sheng Zhang, Liang-qing Zhao, Qian-qian Ling-hu, Gang Xu

**Affiliations:** 1 School of Chinese Ethnic Medicine, Guizhou Minzu University, Guiyang, 550025 Guizhou China; 2 School of Chinese Medicinal Resource, Guangdong Pharmaceutical University, Guangzhou, 510006 Guangdong China; 3 School of Life Sciences/Key Laboratory of Plant Resource Conservation and Germplasm Innovation in Mountainous Region (Ministry of Education), Guizhou University, Guiyang, 550025 Guizhou China; 4 Guizhou Botanical Garden, Guiyang, 550005 China

**Keywords:** Floral development, *GASA* family, *JcGASA6*

## Abstract

**Background:**

The gibberellic acid-stimulated Arabidopsis (*GASA*) gene encodes a class of cysteine-rich functional proteins and is ubiquitous in plants. Most GASA proteins are influence the signal transmission of plant hormones and regulate plant growth and development, however, their function in *Jatropha curcas* is still unknown.

**Results:**

In this study, we cloned JcGASA6, a member of the *GASA* family, from *J. curcas*. The JcGASA6 protein has a GASA-conserved domain and is located in the tonoplast. The three-dimensional structure of the JcGASA6 protein is highly consistent with the antibacterial protein Snakin-1. Additionally, the results of the yeast one-hybrid (Y1H) assay showed that *JcGASA6* was activated by Jc*ERF1*, Jc*PYL9*, and JcFLX. The results of the Y2H assay showed that both JcCNR8 and JcSIZ1 could interact with JcGASA6 in the nucleus. The expression of *JcGASA6* increased continuously during male flower development, and the overexpression of *JcGASA6* was associated with filament elongation of the stamens in tobacco.

**Conclusion:**

*JcGASA6*, a member of the *GASA* family in *J. curcas*, play an important role in growth regulation and floral development (especially in male flower). It is also involved in the signal transduction of hormones, such as ABA, ET, GA, BR, and SA. Also, JcGASA6 is a potential antimicrobial protein determined by its three-dimensional structure.

**Supplementary Information:**

The online version contains supplementary material available at 10.1186/s12870-023-04067-4.

## Background

The gibberellic acid-stimulated Arabidopsis (*GASA*) gene family is a plant-specific group of genes. This family comprises many genes, and most members are regulated by gibberellin (GA). By far, a great number of *GASA* genes have been isolated and identified from *Populus trichocarpa*, *Glycine max*, *Arabidopsis*, and *Solanum tuberosum* [1–4]. The structure and function of this kind of proteins are deeply understood by the analysis of different GASA members identified from a variety of plant species. GASA protein family is a class of cysteine-rich functional proteins. They all have a highly conserved GASA domain (marked by 12 cysteines), which is essential for their normal function [1, 2].

The GASA proteins regulate plant growth and development, including seed germination, lateral root formation, stem elongation, flowering, flower and fruit development, biotic and abiotic stress responses, and signal transduction of hormones [5–9]. The functions of the genes in the *GASA* family have been mostly studied in *Arabidopsis*. Most GASA proteins are involved in hormone signal transduction. *GAST1*, *GASA4*, *GASA6*, *GASA9* and *GASA14* are involved in gibberellin signal transduction and are located downstream of DELLA protein [5, 10]. The GASA proteins are also involved abscisic acid (ABA) signal transduction. The expression of *NtGASA1*, *NtGASA2*, *NtGASA9* and *AtGASA14* can be induced by ABA [5, 11]. However, no study has shown that proteins in the GASA family can perform ethylene (ET) signal transduction, although crosstalk between ET and gibberellin is common in seed germination, stem elongation, and flowering [12, 13]. Several GASA proteins also regulate flower development. For example, the overexpression of *AtGASA5* causes delayed flowering, whereas the mutation of *AtGASA5* leads to early flowering [14]. Functional deletions in *AtGASA6* and *AtGASA4* were found to cause late flowering, but early flowering was caused by only the overexpression of *AtGASA6* [6].

*Jatropha curcas* (Euphorbiaceae) is widely distributed in tropical and subtropical areas and is an ideal bioenergy crop for its oil-rich seeds that are especially rich in unsaturated fatty acids [15, 16]. However, due to low seed yield, the economic benefits of further expanding the *Jatropha*-based biodiesel industry are limited. The low ratio of female to male flowers (1:10–1:30) is a critical factor attributed to the low seed yield of *J. curcas* [15, 17]. The development of the flower of *J. curcas* has received much attention. In another study, using transcriptome data, we found that *JcGASA6,* a member of the *GASA* family, is differentially expressed in the flower buds during the development of the flowers of *J. curcas* [18]. However, the *JcGASA6* functions have not been further investigated.

In this study, *JcGASA6* was cloned from *J. curcas* and expressed in vitro. Upstream regulators of *JcGASA6* and interactive proteins with JcGASA6 protein have been evaluated and screened. Moreover, we observed the expression pattern of *JcGASA6* at several key stages during flower development and further analyzed the phenotypic of overexpression *JcGASA6* in tobacco. Our results clarified the function of *JcGASA6* from the protein and gene levels, and also enriched the signal network of GASA protein in hormone crosstalk.

## Results

### Bioinformatics analysis and subcellular localization of JcGASA6 protein

**Fig. 1:**
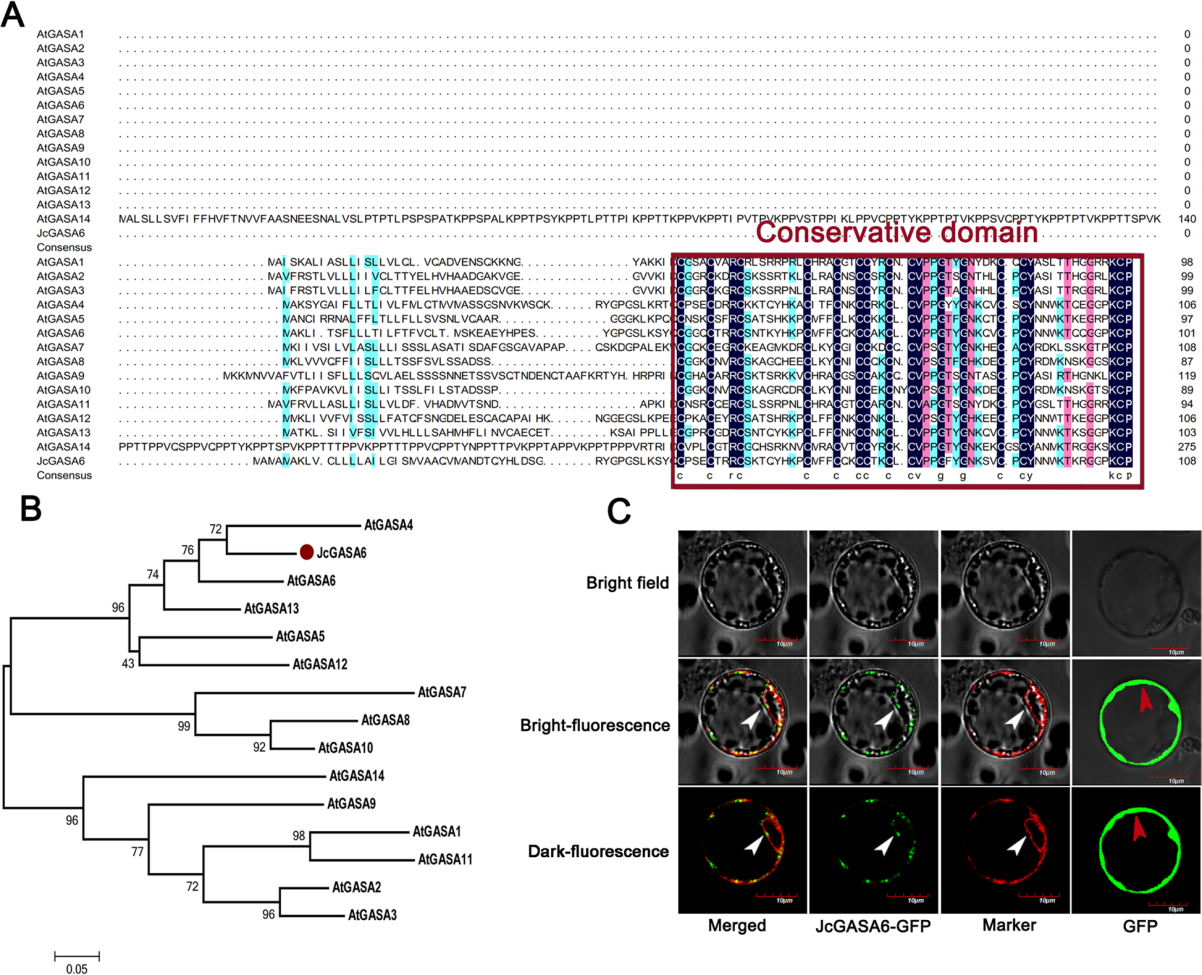
Amino acid sequence alignment analysis and subcellular localization of JcGASA6 protein. **A** Multiple amino acid sequence alignment of JcGASA6 with GASA family. **B** Phylogenetic analysis of the JcGASA6 protein. **C** The subcellular localization of JcGASA6 protein. Red arrow indicates the cell membrane, and white arrow indicates the tonoplast

We obtained the coding sequence (CDS) of *JcGASA6* from cDNA library of flower bud. The CDS of *JcGASA6* contains 327 bp and encodes 108 amino acids. The start codon is ATG and the stop codon is TAG (Fig. S1). Amino acid sequence alignment between JcGASA6 and 14 members of the GASA family from *Arabidopsis* show that JcGASA6 protein has a special sequence composed of 12 cysteines (C-3X-C-2X-RC-8X-C-3X-C-2X-2C-2X-C-X/2X-CV-2X-G-2X-G-4X-C-X/2X-CY-10X-KCP). This special sequence is a conserved domain of GASA family (Fig. 1A). The phylogenetic tree of protein sequences between JcGASA6 protein and Arabidopsis GASA family indicated that JcGASA6 protein is most closely related to AtGASA4 protein sequences (Fig. 1B). The first 24 amino acids at the N-terminal of JcGASA6 protein were the putative signal peptides according to UniProt (Fig. S2). This suggests that JcGASA6 may transfer after synthesis. Further study of the localization of JcGASA6 protein. Under the excitation of 480 nm, the results of laser confocal microscopy showed that the control protein (pBWA(V)HS-GFP) could emit light normally and located in the cell membrane, while the fusion protein (pBWA(V)HS-*GASA6*-GFP) could emit light in the tonoplast besides the cell membrane. Co-localization of the marker protein in tonoplast and the fusion protein showed that green and red fluorescence could be detected at tonoplast (Fig. 1C). This indicated that JcGASA6 protein was located in the tonoplast.

### Expression and identification of *JcGASA6* in vitro

**Fig. 2:**
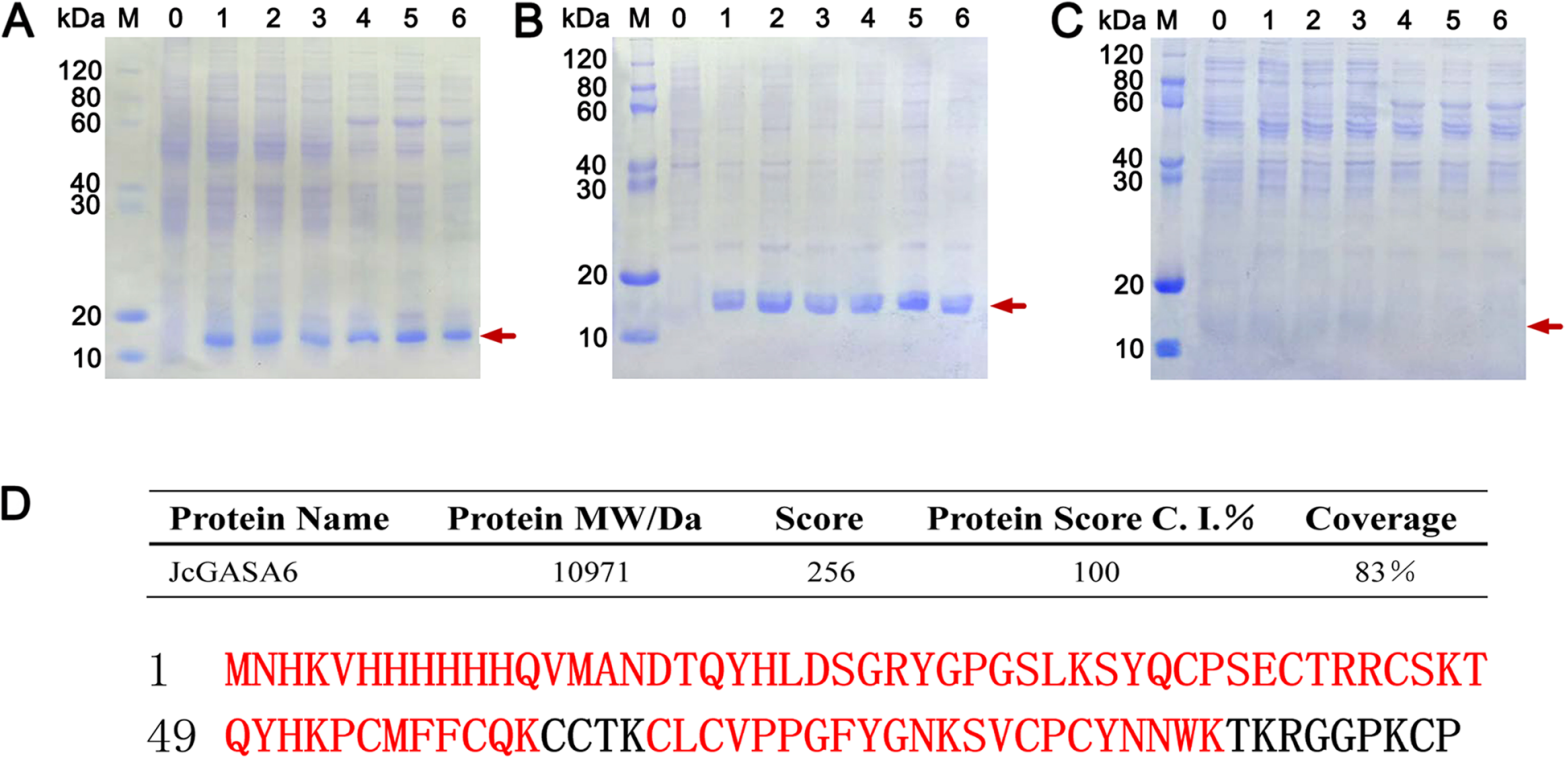
Prokaryotic expression and identification of *JcGASA6*. **A** Whole bacteria SDS-PAGE analysis of *JcGASA6* expression. **B **SDS-PAGE analysis of precipitation from bacteria. **C** SDS-PAGE analysis of supernatant from bacteria. M, marker; lane 0, control (empty pColdII); lane 1–3, expression of *JcGASA6* in BL21(DE3); lane 4–6, expression of *JcGASA6* in ESLa; lane 1and 4, induction with 0.10 mM IPTG for 16 hours; lane 2 and 5, induction with 0.25 mM IPTG for 16 hours; lane 3 and 6, induction with 0.25 mM IPTG for 4 hours; red arrow indicates JcGASA6 protein. **D** Mass spectrometry identification results of expression product from *JcGASA6*

To further study *JcGASA6* from the protein level, *JcGASA6* was cloned into pColdII vector, then was overexpressed in BL21 and ESLA to get JcGASA6 protein. *JcGASA6* encodes a total of 108 amino acids and the signal peptide consists of the first 24 amino acids. Therefore, the molecular weight of the recombinant protein JcGASA6 was 10.97 kDa after adding the tag sequence of vector and removing the signal peptide. SDS-PAGE analysis showed that the expression products from BL21 (with pColdII-*JcGASA6*) or ESLA (with pColdII-*JcGASA6*) contain a 10.97 kDa protein compared with the control (Fig. 2A). This indicates that *JcGASA6* was successfully expressed in BL21 and ESLA. Additionally, the 10.97 kDa protein was distributed in the precipitates released from *E.coli* rupture (Fig. 2B) and was not detected in the supernatants of *E.coli* rupture (Fig. 2C). This indicates that *JcGASA6* was expressed in inclusion bodies way. The 10.97 kDa protein was further identified by ESI-LC-MS/MS. The results showed that a total of 17 peptides from the 10.97 kDa protein matched the amino acid sequence of JcGASA6 protein (Table 1) and the coverage of amino acids match with JcGASA6 protein reached 83% (Fig. 2D). Therefore, the 10.97 kDa protein is JcGASA6 protein.

**Table 1:** Seventeen peptides matched with amino acid sequence of JcGASA6

No.	position	Matched peptide sequence	MW(Da)		Error rate/Da
			Calculation	Observation	
1	1–26	MNHKVHHHHHHQVMANDTQYHLDSGR	3165.4383	3166.4219	−0.0237
2	27–43	YGPGSLKSYQCPSECTR	1988.8720	1989.8728	−0.0066
3	34–43	SYQCPSECTR	1230.4878	1230.4884	0.0006
4	34–43	SYQCPSECTR	1287.5093	1287.5027	−0.0066
5	34–43	SYQCPSECTR	1287.5093	1287.5027	−0.0066
6	34–44	SYQCPSECTRR	1386.5889	1386.574	−0.0149
7	34–44	SYQCPSECTRR	1443.6104	1443.6012	−0.0092
8	34–44	SYQCPSECTRR	1443.6104	1443.6012	−0.0092
9	34–44	SYQCPSECTRR	1329.5675	1329.5389	−0.0286
10	48–60	TQYHKPCMFFCQK	1717.7648	1717.7571	−0.0077
11	48–60	TQYHKPCMFFCQK	1774.7863	1774.7654	−0.0209
12	48–60	TQYHKPCMFFCQK	1774.7863	1774.7654	−0.0209
13	48–60	TQYHKPCMFFCQK	1790.7812	1790.7656	−0.0156
14	65–76	CLCVPPGFYGNK	1410.6424	1411.6293	−0.0205
15	77–86	SVCPCYNNWK	1270.5344	1270.5083	−0.0261
16	77–86	SVCPCYNNWK	1327.5559	1327.5404	−0.0155
17	77–86	SVCPCYNNWK	1327.5559	1327.5404	−0.0155

**Fig. 3:**
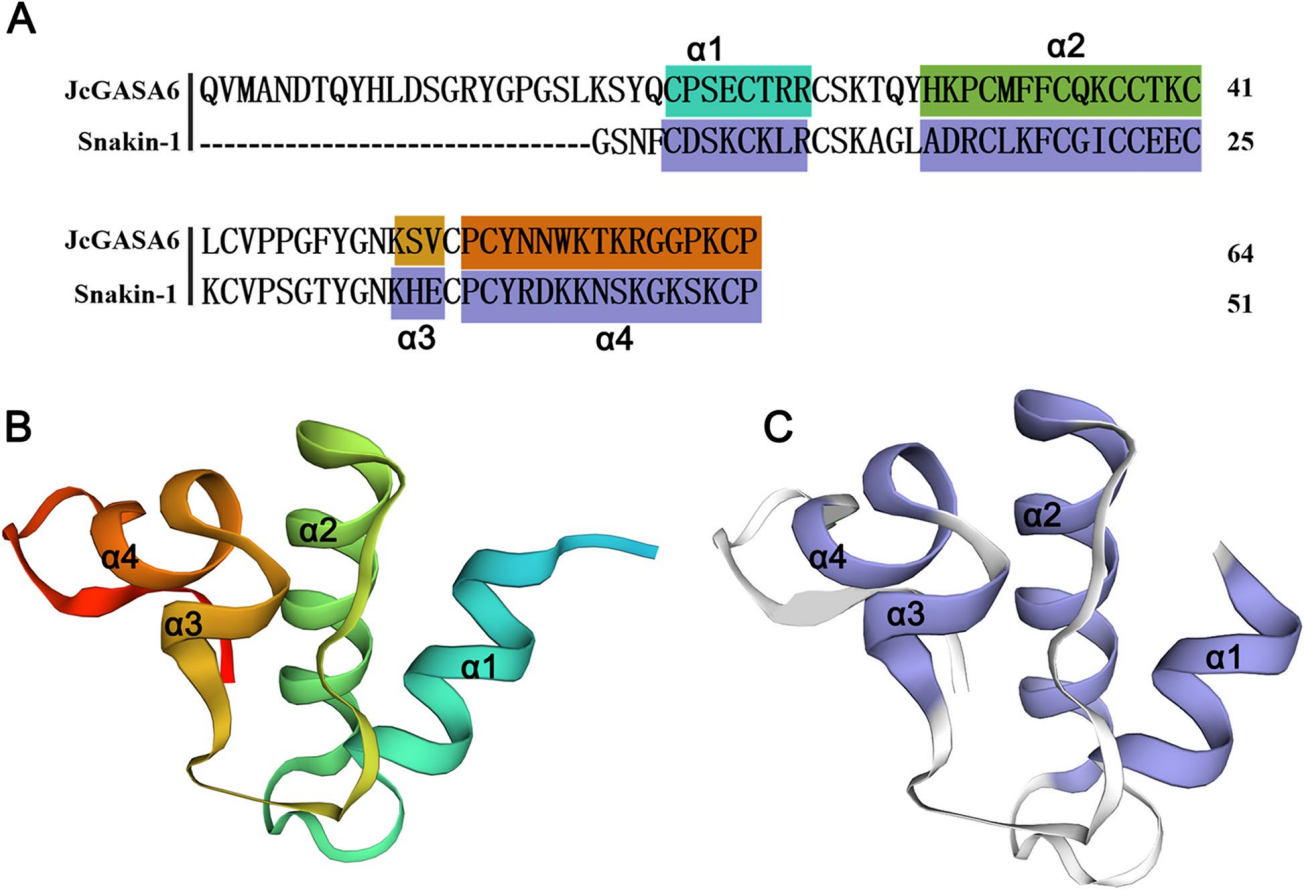
Comparison of JcGASA6 protein and Snakin-1 protein. **A** Amino acid sequence alignment of JcGASA6 protein and Snakin-1 protein. **B** Speculative three-dimensional structure of JcGASA6 protein. **C** Three-dimensional structure of Snakin-1 protein

JcGASA6 protein and Snakin-1 protein (crystal 5e5t.1A) have the highest homology according to the results from the SWISS-MODEL database. Therefore, taking the crystal of 5e5t.1A as the template, we get the speculative three-dimensional structure of JcGASA6 protein after homologous modeling. Both JcGASA6 protein and Snakin-1 protein have two important structures-short helices. The first short helix is composed of α1 and α2, and the second short helices is composed of α3 and α4 (Fig. 3A). These two structures are essential for the function of GASA family protein [19]. Moreover, like Snakin-1 protein, JcGASA6 protein contains 12 cysteines. The 12 cysteine residues form 6 disulfide bonds (Fig. 3B, C), which is of great significance to maintaining the stability of protein three-dimensional structure [20].

### Promoter sequence characteristics and upstream regulatory factors of *JcGASA6*

**Fig. 4:**
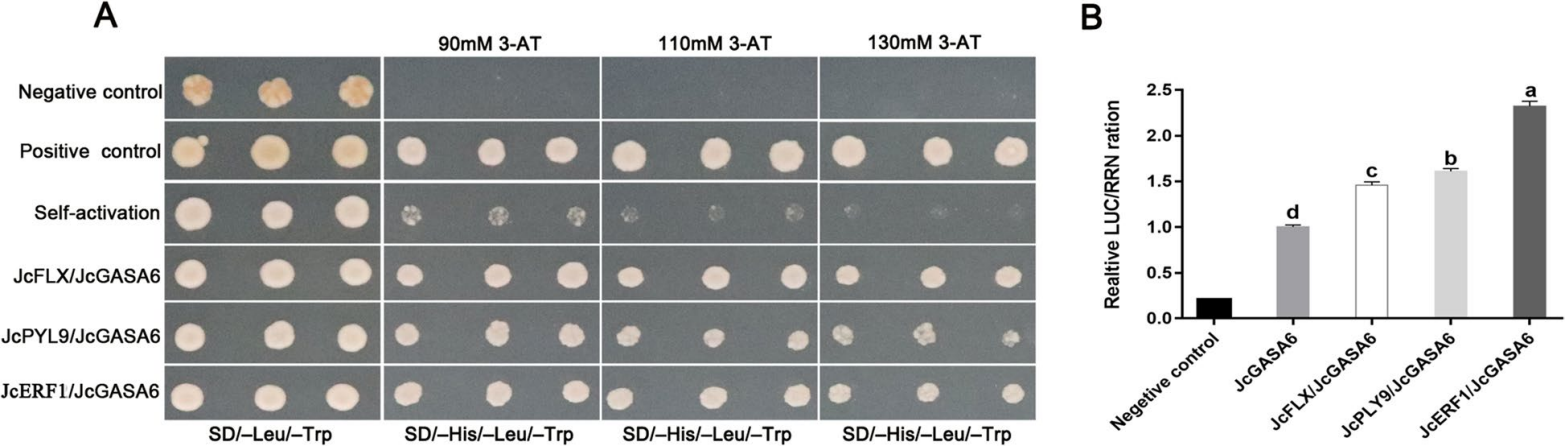
All of JcFLX, JcPYL9 and JcERF1 binds to the promoter of *JcGASA6* and activate its expression. **A** Yeast one-hybrid assay shows all of JcFLX, JcPYL9 and JcERF1 bind to *JcGASA6* promoter. **B** Dual-luciferase assays showed all of JcFLX, JcPYL9 and JcERF1 can activate the expression of *JcGASA6*. Diverse lowercase letters indicate significant differences (*P* < 0.05). The significance of difference was analyzed by Tukey’s test

To further reveal the potential signal transduction pathway involved by *JcGASA6*, the promoter of *JcGASA6* was isolated from *J. curcas* genome DNA. Except for conserved motifs (AT-TATATA-box, CAAT-box, and TATA-box), *JcGASA6* promoter included several motifs with unknown function. Additionally, *JcGASA6* promoter also included an ethylene-responsive motif which suggested the expression of *JcGASA6* may be regulated by ET (Fig. S3). Then, the upstream regulatory factors of *JcGASA6* were screened by yeast one-hybrid (Y1H) system using *JcGASA6* promoter as bait. Three genes, which may bind to the promoter of *JcGASA6*, were screened from cDNA library of *J. curcas* flower. The three genes included *JcFLX*-like (ID: XM_012232748.2), *JcERF1* (ID: XM_012213416.2), and *JcPYL9* (ID: XM_012227842.2). To confirm that the three genes can bind to the promoter of *JcGASA6*, the three genes were tested by Y1H individually. Self-activation of promoters is common, so 3-amino-1,2,4-triazole (3-AT) with suitable concentration is used to inhibit the self-activation of promoters [21]. In our study, 3-AT with a concentration of 130 mM completely inhibit the self-activation of *JcGASA6* promoter (Fig. 4A). Additionally, the yeast cells, which contain pGADT7-*JcFLX* and *JcGASA6*-Pro-pHis2, pGADT7-*JcPYL9* and *JcGASA6*-Pro-pHis2, or pGADT7-*JcAP2* and *JcGASA6*-Pro-pHis2, can grow normally on SD/−His-Leu-Trp medium with 130 mM 3-AT (Fig. 4A). These results indicated that all of JcFLX, JcPYL*9*, and JcERF1 could interact with *JcGASA6* promoter to regulate the expression of *JcGASA6*. Furthermore, the regulation of three genes on *JcGASA6* was determined by dual-luciferase assays. The results of dual-luciferase assays showed that all of JcFLX, JcPYL9, and JcERF1 could enhance the activity of Luc driven by *JcGASA6* promoter and the activity of LUC was the strongest in *JcERF1*/*JcGASA6* group (Fig. 4B). This suggested that all of the three genes (*JcFLX*, *JcPYL9* and *JcERF1*) could interact with *JcGASA6* promoter to activate the expression of *JcGASA6*, and *JcERF1* has the strongest activation effect on *JcGASA6*.

### Proteins interacting with JcGASA6 protein

**Fig. 5:**
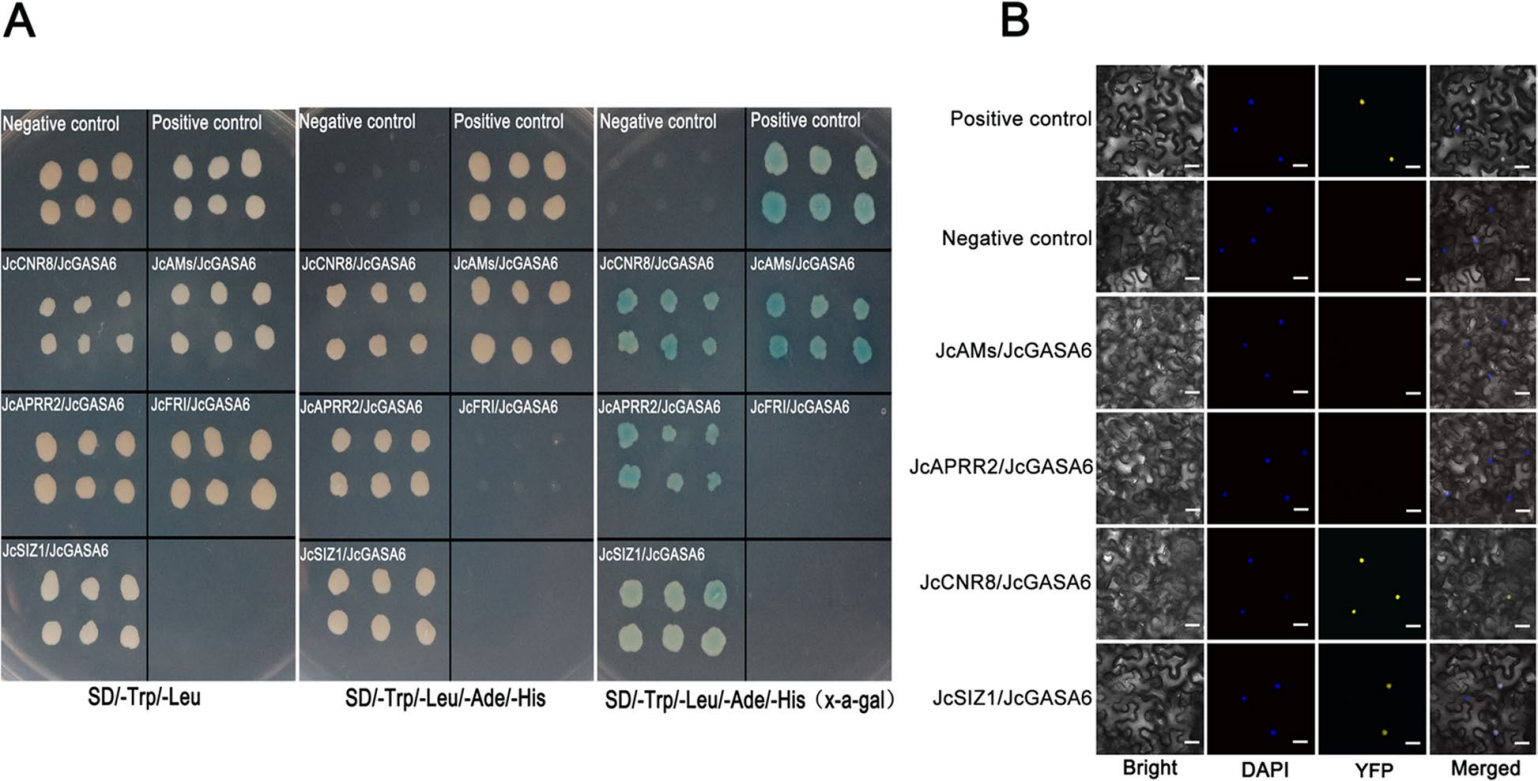
Identification of proteins interacted with JcGASA6. **A** Y2H assay to verify the interactions of JcGASA6 with JcCNR8, JcAMs, JcAPRR2, JcFRI and JcSIZ1. **B** BiFC assay of the interactions of JcGASA6 with JcAMs, JcAPRR2, JcCNR8 and JcSIZ1; Bright: bright field without fluorescence signal; NLS: blue fluorescent signal located in the nucleus; YFP: yellow fluorescent signal of interacting proteins; Merged: Overlap of yellow and blue fluorescence signals in bright field, Bars: 48 μm

In addition to screening the upstream regulators of *JcGASA6*, we also screened the interacting proteins of JcGASA6 protein by using yeast two-hybrid (Y2H) system. On SD/−Ade/−His/−Leu/−Trp medium, the yeast cells with pGBKT7–53 and pGADT7-T plasmids can grow normally, while the yeast cells with pGBKT7 and pGADT7 plasmids (negative control) and yeast cells with pGBKT7-*JcGASA6* and pGADT7 plasmids can not grow normally (Fig. S4). These results indicated that JcGASA6 protein without transcriptional self-activation activity. JcGASA6 was used as the bait to screen its interacting proteins from flower bud cDNA library, and five proteins interacting with JcGASA6 were obtained. The five proteins include JcCNR8 (*J. curcas* cell number regulator 8, ID: XM_012226926.2), JcAMs (*J. curcas* transcription factor ABORTED MICROSPORES, ID: XM_020682349.1), JcAPRR2 (*J. curcas* two-component response regulator-like APRR2, ID: XM_012218861.2), JcFRI (*J. curcas* FRIGIDA-like protein 4a FRI, ID: XM_012214239.2) and JcSIZ1(*J. curcas* E3 SUMO-protein ligase SIZ1, ID: XM_012209470.2). To verify the interaction between the five proteins with JcGASA6, we used Y2H assay. Yeast cells, which containing JcCNR8 and JcGASA6, JcAMs and JcGASA6, JcAPRR2 and JcGASA6 or JcSIZ1 and JcGASA6, grew normally on SD/−Trp/−Leu/−Ade/−His and SD/−Trp/−Leu/−Ade/−His (X-a-gal) medium, while the yeast cells with JcFRI and JcGASA6 could not grow (Fig. 5A). These results suggested four proteins JcCNR8, JcAMs, JcAPRR2, and JcSIZ1 may interact with JcGASA6 protein. BiFC assay was carried out to further confirm these results. The BIFC assay show in Fig. 5B, the yellow fluorescence could be detected only when the protein pair of JcCNR8 and JcGASA6 or JcSIZ1 and JcGASA6 were transiently co-expressed in leaf epidermal cells, and the yellow fluorescence was detected in the nucleus (Fig. 5B). These results indicated that both JcCNR8 and JcSIZ1 could interact with JcGASA6 in nucleus.

### Expression pattern of *JcGASA6* in the flower development of *J. curcas*

**Fig. 6:**
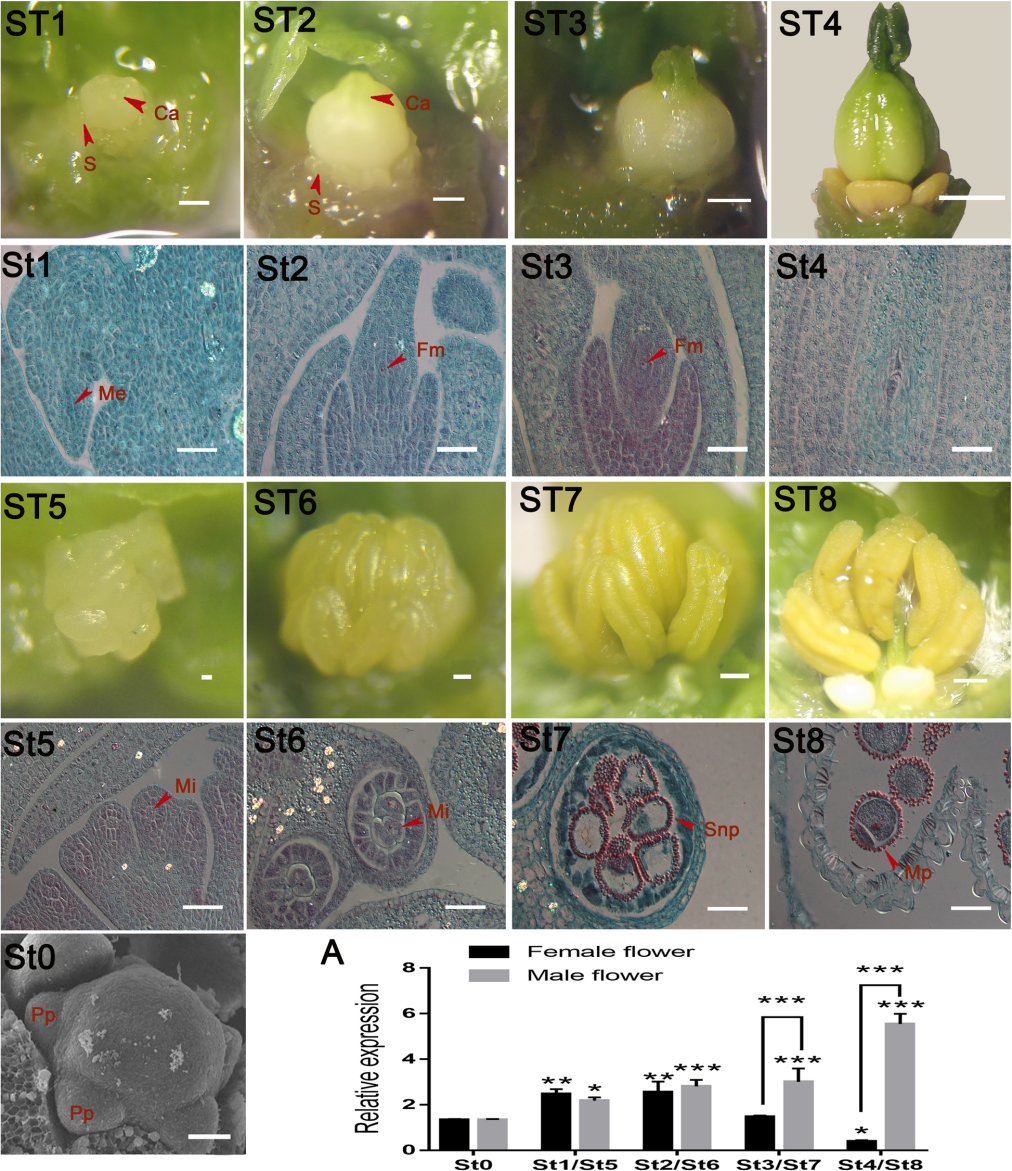
Morphological and histological observation on flower of *J. curcas.* ST1 to ST4 represent the morphology of female flower and the corresponding histological structures are St1 to St4. ST5 to ST8 represent the morphology of male flower, and the corresponding histological structures are St5 to St8. St0, represent the morphology of undifferentiated flower. **A** Expression profile of *JcGASA6* during the development of flower, asterisks indicate a significant difference, *p < 0.05, **p < 0.01, ***p < 0.001. Ca, carpel; Pp, petal primordium; S, stamen; Me, megasporocyte; Fm, functional megaspore; Mes, mononuclear embryo sac; Mi, microsporocyte; Snp, single nucleus pollen; Mp, mature pollen. Bars = 50 μm for histological structures of flower. Bars = 270 μm for morphology of female flower. Bars = 20 μm in St0

Our previous studies suggest that *JcGASA6* may also be involved in flower development [18], we further analyzed the expression pattern of *JcGASA6* during flower development. In order to accurately judge the expression pattern of *JcGASA6* during flower development of *J. curcas*, the flower of *J. curcas* was observed at the morphological and histological. When the bud length of *J. curcas* is about 0.15 mm, the primordium of petal has appeared, but the sexual differentiation of flower has not yet begun (Fig. 6St0). During the development of female flowers, the carpel did not fully heal when the ovary length is about 0.80 mm (Fig. 6ST1). Histological analysis showed that the female flower was at the stage of megasporocyte (Fig. 6St1). After that, the megasporocyte undergoes two meiosis to form functional megaspore (Fig. 6ST2), at this time, the carpel has basically healed (Fig. 6St2), and the ovary length was about 1.10 mm. The functional megaspore develops further and enters the mononuclear embryo sac stage (Fig. 6St3), the ovary length was about 1.50 mm (Fig. 6ST3). Finally, functional megaspores formed mature embryo sac with 8-core 7 cells through three mitoses (Fig. 6St4), and the ovary length was about 3.20 mm (Fig. 6ST4). During the development of male flower, the male flower was in the stage of microspore mother cell when the flower bud length is about 0.50 mm (Fig. 6St5, ST5). After two times of meiosis, the microsporocyte entered the tetrad stage (Fig. 6St6), the length of bud was 1.20 mm (Fig. 6ST6). Microsporocyte was released to the anther chambers to form single nucleus pollen (Fig. 6St7), the length of the bud was 2.00 mm (Fig. 6ST7). Finally, single nucleus pollen undergoes nuclear division to form mature pollen with two-cell (Fig. 6St8), the length of bud was about 3.00 mm (Fig. 6ST8). In female flowers, the expression of *JcGASA6* gradually increased from the undifferentiated stage (St0) to the megasporocyte meiosis stage (St2) and reached the highest lever at the megasporocyte meiosis stage. After that, the expression of *JcGASA6* gradually decreased and reached the lowest level at the mature embryo sac stage (St4). These results suggested that *JcGASA6* plays an important role in the early development of female flower. In male flowers, the expression of *JcGASA6* increased from undifferentiated stage (St0) to mature pollen stage (St8) and reached the highest level at mature pollen stage. These results indicate that *JcGASA6* plays an important role in the whole development of male flower. Moreover, the expression of *JcGASA6* was not significantly different in the early development between female (St1-St2) and male flower (St5-St6), while in the late development of female (St3-St4) and male flowers (St7-St8), the expression of *JcGASA6* increased significantly in male flowers. This suggested that *JcGASA6* was more important for the development of male flower than female flower (Fig. 6A).

### Overexpression of *JcGASA6* promoted the elongation of stamen filament in *tobacco*

**Fig. 7:**
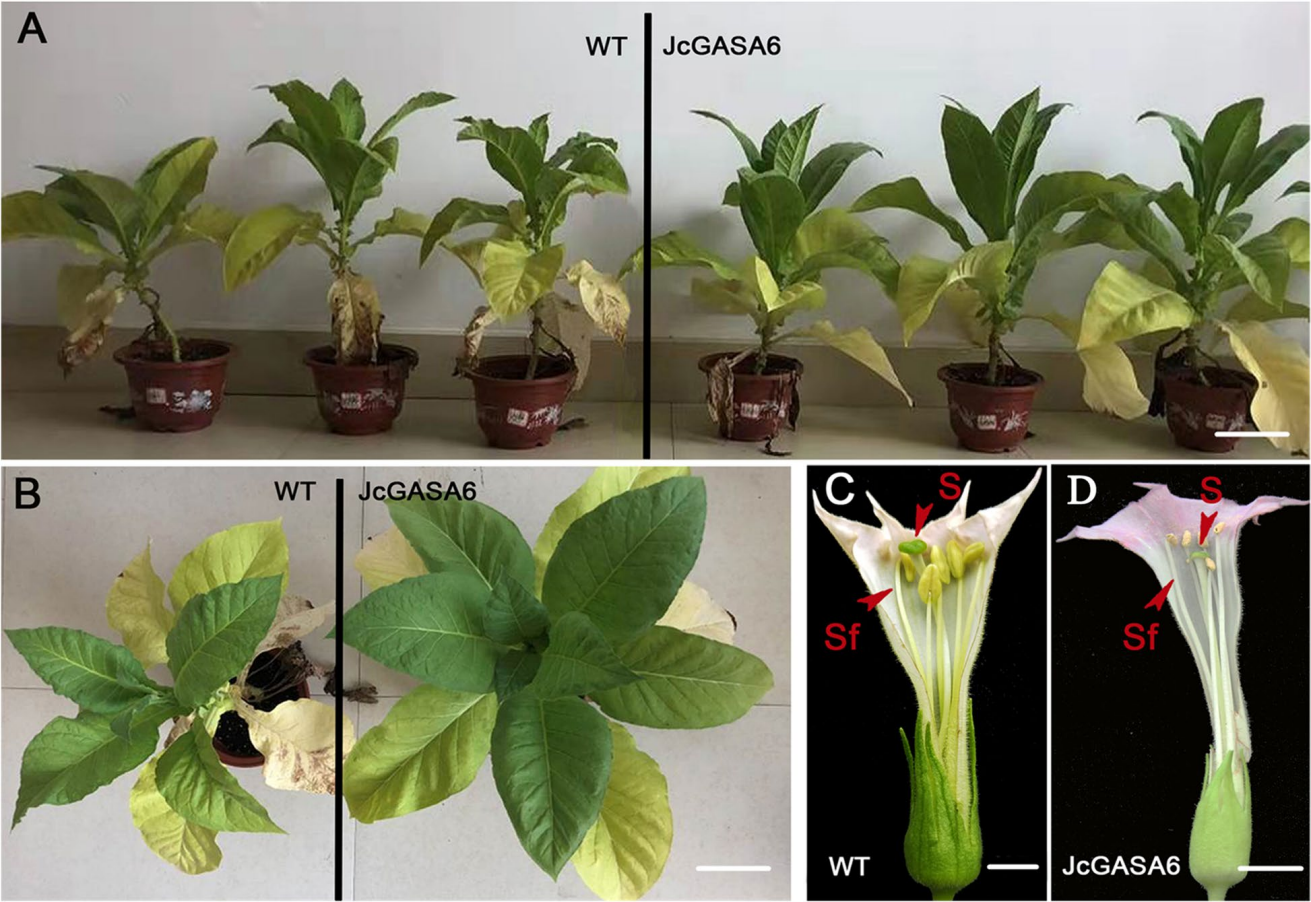
Phenotype of tobacco overexpressing *JcGASA6*. **A** and **B** plant shape of wild-type tobacco and transgenic tobacco. **C** The flower of wild-type tobacco. **D** The flower of transgenic tobacco. Sf, stamen filament, S, stigma. Bars = 1.8 cm in **B**, **C**, **D**. Bars = 7.5 cm in **A**

The expression pattern of *JcGASA6* during flower development suggests that *JcGASA6* plays an important role in flower development, especially in male flower development. To further reveal the role of *JcGASA6* in flower development, overexpression of *JcGASA6* in tobacco (wild-type *tobacco* were controls, WT). There was no significant difference in plant height, ground diameter and style length between wild-type tobacco and transgenic tobacco (Table 2). However, the stamen of transgenic tobacco (TR) was higher than the stigma of ovary, and the filament length of TR tobacco was longer than WT tobacco (Table 2 and Fig. 7C, D). This result suggested that *JcGASA6* could promote the elongation of stamen filament. Moreover, the leaves of TR tobacco are longer and wider than those of WT tobacco (Table 2 and Fig. 7A, B). This result indicated that *JcGASA6* was also involved in plant growth.

**Table 2:** Comparison of growth and development between wild-type tobacco and transgenic tobacco

Sample	Plant height (cm)	Ground diameter (mm)	Leaf length (cm)	Leaf width (cm)	Style length (cm)	Filament length (cm)
**WT**	29.18 ± 1.52a	9.79 ± 0.38a	24.00 ± 0.36a	12.59 ± 0.06a	5.31 ± 0.03a	4.93 ± 0.06a
**TR**	29.28 ± 1.64a	10.00 ± 0.51a	31.23 ± 0.37b	15.40 ± 0.38b	5.31 ± 0.03a	5.61 ± 0.07b

Different lowercase letters in each column indicate the significance between wild-type tobacco and transgenic tobacco (*P* < 0.05)

## Discussion

Gibberellic Acid-stimulated Arabidopsis (GASA) protein, also known as Snakin protein, is a kind of CRP (cysteine-rich peptides) protein [8]. GASA family proteins have been found in many plants and have many members, characterize by a conserved domain containing 12 cysteines [11]. In present study, *JcGASA6* encoded a protein identified as JcGASA6 protein. JcGASA6 is a member of GASA family, suggested by its typical domain containing 12 cysteines (Fig. 1A). It was with a length of 108 amino acids and a molecular weight of 10.97 KD, containing a signal peptide at N-terminal. It showed the closest genetic relationship with *AtGASA4* (Fig. 1B).

Antimicrobial peptides (AMPs) are excellent candidate drugs against drug-resistant pathogens. The structure of AMPs, especially an α-helical hairpin structure, plays an important role in killing pathogens [20]. Snakin is the only GASA member with antibacterial effect [3, 22]. Two short helices structures (dbHTH) in Snakin-1 protein are similar to the α-helical hairpin in antimicrobial peptide EcAMP1 [23]. The short helices and six disulfide bonds can form a large positive electrostatic surface (Fig. 3C). The positive electrostatic surface makes Snakin-1 protein play an antibacterial role [20]. Similar to the Snakin-1 protein, the speculative three-dimensional structure of JcGASA6 protein have two important structures-short helices and six disulfide bonds (Fig. 3), which might endow it with an antibacterial role.

Protein localization is often closely related to its function. Most GASA proteins have signal peptides at the N-terminal, and they are located in the plasma membrane or cell wall. AtGASA14 protein is located in the plasma membrane which will make it easier to regulate the balance of reactive oxygen [5]. GhGASA10 (a GASA protein in Cotton) was localized in the plasma membrane, which conducive to synthesize proteins in the cell wall to promotes the cell elongation in fiber [24]. AtGASA6 protein is located in the cell wall, which helps to regulate the elongation of hypocotyl and seed germination [13]. In our study, The N-terminal of JcGASA6 protein also has a signal peptide (Fig. S3). But unlike other members of the GASA protein, JcGASA6 is located at the tonoplast (Fig. 1C). This suggests that the function of JcGASA6 protein may be different from other members of GASA family.

GASA family is firstly thought to play important role in gibberellin signal pathway. *AtGASA6* and *AtGASA4* are located the downstream of DELLA protein in GA signal pathway [10]. To data, most members of the *GASA* family are found to be widely involved in hormone signal transduction, such as ABA, ET, GA, BR (brassinolide), SA (salicylic acid). *OsGSR1* control BR biosynthesis to regulate salt stress [25]. *Snakin-1* is involved in hormonal balance and *Snakin-1* silencing enhanced GA and SA levels [26]. *AtGASA6* is down-regulated by ABA and can integrate GA and ABA to promote cell elongation and seed germination [13]. Our study also found that ABA receptor JcPYL9 protein could directly activate the expression of *JcGASA6* (Fig. 4), supporting that the members of GASA family would be involved in ABA signal transduction. A few evidence showed that members of GASA protein family were involved in ET signal transduction. *FaGAST2*, a member of GASA family in strawberry, was up-regulated by ethephon [7]. ERF1 is the target gene of the core transcription factor EIN3 of the ET signaling pathway. EIN3 protein can activate the expression of *ERF1* through binding to the promoter of *ERF1* [27]. Our results showed that the expression of *JcGASA6* could be activated by ERF1 protein in *J. curcas* (Fig. 4), which further confirmed that GASA protein family is involved in ET signal transduction.

AtGASA4 (closeted genetic relationship with JcGASA6 protein) and AtGASA6 (the homologous protein of JcGASA6) are involved in flower development. The *gasa4* mutant showed more leaf buds before generating flowers, but overexpression of *AtGASA4* could not promote flowering transformation. This indicates that *AtGASA4* is not enough to induce flowering transformation [28]. *AtGASA4* and *AtGASA6* also affect flowering time, but *AtGASA6* plays a predominant role in causing early flowering [6]. *FLX* is reported to be involved in the regulation of early flowering [29]. In present study, FLX could directly activate the expression of *JcGASA6.* Although we did not observe abnormal flowering in transgenic tobacco, overexpression of *JcGASA6* caused elongated filaments in tobacco (Figs. 6 and 7C). Furthermore, the expression of *JcGASA6* increased continuously during male flower development. All these results suggested that *JcGASA6* would contribute to the development of male flowers, especially the filaments of stamens. This affection of *JcGASA6* on flower is different from other *GASA* family (such as *AtGASA4* and *AtGASA6*). On the other hand, JcGASA6 protein could interact with JcCNR8 protein. CNR can control organ size by regulating cell number [30]. These would explain that the leaf size of transgenic tobacco was larger than wild-type tobacco (Fig. 7A, B) (Table 3). Therefore, *JcGASA6* is also involved in growth regulation. Also, this is the first time to find that the GASA family can interact with CNR protein, which may support *AtGASA10* facilitate wall growth [4].

**Table 3:** Parameters of developmental stages of *J. curcas* flower

Developmental stage	Flower organ size/mm
Undifferentiated stage (St0)	0.15 ± 0.01
♀ Megasporocyte stage (St1)	0.80 ± 0.03
♀ Megasporocyte meiosis stage (St2)	1.00 ± 0.06
♀ Mononuclear embryo sac stage (St3)	1.50 ± 0.03
♀ Mature embryo sac stage (St4)	3.20 ± 0.04
♂ Microsporocyte stage (St5)	0.50 ± 0.01
♂ Tetrad stage (St6)	1.20 ± 0.03
♂ Single nucleus pollen stage (St7)	2.00 ± 0.05
♂ Mature pollen stage (St8)	3.00 ± 0.05

Additionally, the JcGASA6 protein also could interact with SUMO E3 ligase (JcSIZ1). SUMO (Small Ubiquitin-like Modifier) E3 ligase is an important member involved in protein SUMO modification. SUMO E3 ligase recruits target proteins and promote the binding of SUMO to the target protein, then the activity of the target protein changes reversibly [31, 32]. Therefore, the activity of JcGASA6 protein may be modified by SUMO.

## Conclusions

*JcGASA6*, a member of the GASA family, has an antibacterial structure similar to the antibacterial peptide Snakin-1, and is located in the tonoplast. In addition, JcGASA6 can be activated by the ABA receptor JcPYL9 and the core transcription factor JcERF1, which supports that JcGASA6 can participate in ABA and ET signal transduction. Meanwhile, *JcGASA6* is activated by the early flowering gene *FLX*, and its expression increases continuously during male flower development. Abnormal flowering was not observed after overexpressing *JcGASA6*, but the elongation of stamen filaments was caused by the overexpression of *JcGASA6*. These results confirmed that *JcGASA6* is involved in flower development. Additionally, overexpression of *JcGASA6* increased the leaf size in transgenic plants, and JcGASA6 can interact with CNR (a protein with the regulation cell number). These results indicate that *JcGASA6* is also involved in growth regulation. Moreover, JcGASA6 interacts with ubiquitin ligase SIZ1 also implies that its activity may be modified by SUMO. All in all, our results have revealed the functions of *JcGASA6* from multiple perspectives.

## Methods

### Plant materials and flower collection

The flower buds of *J. curcas L.* were collected from Zhenfeng, Guizhou Province, China (36°14,050.2′N, 87°51,047.8′E). Flower buds for morphological and microscopic observation were temporarily stored in the mixture of acetaldehyde acetic acid 50% alcohol (4: 6: 90, v/v), and for RNA extraction were temporarily stored in RNAlocker (Tiandz, Inc., Beijing China). All samples were placed on ice.

### *JcGASA6* isolation and sequence analysis

The full-length cDNA of *JcGASA6* was cloned from flower bud by RACE-pcr, then the obtained cDNA sequences were aligned in NCBI database (Accession number: KU500008).

Sequence of the GASA family of *Arabidopsis thaliana* was obtained from NCBI database (Table S1). Multiple sequence alignment used DNAMAN, phylogenetic tree constructed by MEGA 6.0, and signal peptide predicted by UniProt. (https://www.uniprot.org/peptidesearch/).

### Subcellular localization, expression and identification of JcGASA6 protein

The full-length cDNA of *JcGASA6* was fused with the pBWA(V)HS-osGFP then recombinant plasmid pBWA(V)HS-JcGASA6-osGFP was transfected transformed into rice protoplasts by PEG (polyethylene glycol). The protoplasts were observed by confocal laser microscope under the excitation of 480 nm wavelength after dark culture at 28 °C for 48 hours (FV10-ASWOLYMPUS, Japan) [33]. Gamatip protein located in the tonoplast was used as a marker [34]. All primers used for subcellular localization are listed in Table S2.

The coding sequence of *JcGASA6* was amplified by specific primers (JcGASA6ex-F and JcGASA6ex-R), then the coding sequence was connected to the expression vector pCold II after digesting with Nde I and Hind III. The sequence of recombinant plasmid JcGASA6-pCold II was examined using vector primers (pCold II-F and pCold II-R) (Table S2). The recombinant plasmid was transformed into *E. coli* competent cells (BL21 or ESLA) to overexpress *JcGASA6*, and the empty vector pCold II was transformed into BL21 or ESLA as the control. Then the BL21 or ESLA were cultured at 16 °C, and isopropyl β-D-thiogalactoside (IPTG) was used as an expression inducer. The details of method same as previously reported [35].

The expression products of *JcGASA6* in *E. coli* were analyzed by SDS-PAGE electrophoresis. Cut off the SDS-PAGE glue containing JcGASA6 protein, and wash the SDS-PAGE glue with ultrapure water and decolorization with acetonitrile mixture. The decolorized SDS-PAGE glue was digested by trypsin overnight at 37 °C to form enzymolysis solution, then the enzymolysis solution was identified by LC-MS/MS method. The method was consistent with the previously reported [36]. The three-dimensional structure of protein was analyzed by the SWISS-MODEL database (https://www.swissmodel.expasy.org/interactive).

### Promoter isolation and analysis, construction of cDNA library for yeast-hybrid system

The promoter sequence of *JcGASA6* was obtained from the published genomic database (ID: 105640538), and PlantCARE was used for promoter sequence analysis (http://bioinformatics.psb.ugent.be/webtools/plantcare/html/). The cDNA library of Yeast-Hybrid System was constructed by mixed RNA from flowers at different developmental stages. The primary library was constructed using attB2 as linker and ATTB-A1, ATTB1-B and ATTB1-C as primers. The clone number of primary librarywas8.04 × 106 cfu. The plasmid of primary library was extracted and transferred into DH10B by the electrotransfer method, and then the secondary library was obtained. The clone number of secondary library was 1.31 × 107 cfu. The method is consistent with that previously reported [37] (Table S3).

### Yeast-one hybrid (Y1H) and dual-luciferase assay

The promoter of *JcGASA6* was amplified using specific primer pro-*JcGASA6*-F/R, then fusion to the pHIS2 (Table S4). Co-transferred fusion plasmid and secondary library to Y187 yeast system, then screen the upstream regulator of *JcGASA6*. The screening process refers to the previous method [38]. Three upstream regulators (*JcFLX*, *JcERF1* and *JcPYL9*) were screened from the secondary library. The interaction between *JcGASA6* promoter and these three regulators was verified one by one with Y187 yeast system [38]. pGADT7 and p53-pHis2 were co-transformed into Y187 as negative controls. pGADT7–53 and p53-pHis2were co-transformed into Y187 as positive controls. The promoter self-activation of *JcGASA6* was detected using Y187 with plasmids pGADT7 and *JcGASA6*-Pro-pHis2.

The primer *JcCASA6*-luc-F/R was used to construct pGreenII 0800-*JcGASA6*-luc, and three primers (AP2-F/R, FLX-F/R and PYL9-F/R) was used to construct regulators (pGreenII 62-*JcFLX*-SK, pGreenII 62-*JcERF1*-SK and pGreenII 62-PYL9-SK). Co-transferred pGreenII 0800-pro*JcGASA6*-luc and regulator into tobacco leaves, then detected the fluorescence value (Dual-Luciferase Assay System, Promega) (Table S4) [38]. pGreenII 62-SK and pGreenII0800-Luc were co-transformed into tobacco leaves as negative controls.

### Yeast-two hybrid(Y2H) and bimolecular fluorescence complementation (BiFC) assay

Full-cDNA *JcGASA6* fusion with pGBKT7 by using GASA6-GBK-F/R primer, then both of pGBKT7-*JcGASA6*and pGADT7-AD were transferred into AH109 by LiAc method, then the AH109was cultured for the detection of self-activation activity. Co-transferredpGBKT7-*JcGASA6* and secondary library to AH109 for screening the interaction proteins of JcGASA6. Five proteins were screened, including JcCNR8, JcAMs, JcAPRR2, JcFRI and JcSIZ1. The interaction between the five proteins andJcGASA6 were verified by one-to-one in AH109 [39] (Table S5). pGADT7 and pGBKT7 were co-transformed into AH109 as negative controls. pGADT7–53 andpGBKT7-T were co-transformed into AH109 as positive controls.

Full-cDNA *JcCNR8*, *JcSIZ1*, *JcAMs*, and *JcAPRR2*were fused with PSPYCE-35Srespectively by using specific primer, and Full-cDNA *JcGASA6*was fused with PSPYNE-35S. Co-transferred PSPYNE-35S-*JcGASA6* and PSPYCE-35S-JcCNR8/PSPYCE-35S-JcSIZ1/PSPYCE-35S-JcAMs/PSPYCE-35S- *JcAPRR2* into EHA105. The five types of EHA105 infected tobacco leaves respectively, then the fluorescence signal was detected 72 hours after infection [39] (Table S5). PSPYCE-35S and PSPYNE-35S were co-transformed into tobacco leaves as negative controls. PSPYCE-35S-bZIP63 and PSPYNE-35S-bZIP63 were co-transformed into tobacco leaves as positive control.

### Morphology and microscopic observation of flower

Flower buds were classified according to their length, dissected under stereoscope, observed and photographed. Since the flower bud of undifferentiated stage is too small to be observed clearly under stereoscope, it was observed under the scanning electron microscope. The undifferentiated flower were fixed in the mixture of formaldehyde-acetic acid-50% ethanol, and then dissected and observed by electron microscope [18]. According to the classification, paraffin sections of these flower buds were made [40] (Table 3).

### Expression of *JcGASA6* during flower development determined by qRT-PCR

The total RNA of flower was extracted by RNA isolation kit (Omega Bio-Tek, Beijing, China), qualified RNA was used to synthesize the first strand cDNA (Takara, Beijing, China). PCR amplification was used Bio-Rad CFX system (Bio-Rad, USA). *Beta-tubulin* and *actin* as internal control. The 2 ^- ΔΔ CT^ method was used to calculate the relative expression of *JcGASA6* [41]. All primers used for PCR amplification are listed in Table S6 and each sample reaction was repeated three times.

### Overexpression of *JcGASA6* in *Nicotiana tabacum L.*

Fusion full-length cDNA of *JcGASA6* with the pBWA(V)KS-GUS. The recombinant plasmid (pBWA(V)KS-*JcGASA6*-*GUS*) was transformed into agrobacterium tumefaciens (GV3101), then positive agrobacterium tumefaciens were screened. Tobacco (K326) leaves infected by GV3101 were cultured in dark at 25 °C for 2 days. After tobacco leaves were differentiated into seedlings, positive tobacco was detected (Fig. S5), and wild-type tobacco was used as control [42] (Table S6).

## Supplementary Information

**Additional file 1.**

**Additional file 2.**

## Data Availability

All data generated or analyzed in our study are available in this article and its supplementary information files. Gene sequences can be down-loaded at NCBI database (https:// www. Ncbi. nlm. Nih. gov/). The GenBank accession number of JcGASA6 is KU500008, AtGASA1 is P46689.2, AtGASA2 is P46688.1, AtGASA3 is P46687.1, AtGASA4 is P46690.2, AtGASA5 is Q84J95.1, AtGASA6 is Q6NMQ7.1, AtGASA7 is O82328.1, AtGASA8 is O80641.1, AtGASA9 is Q8GWK5.1, AtGASA10 is Q8LFM2.1, AtGASA11 is F4IQJ4.1, AtGASA12 is Q6GKX7.1, AtGASA13 is A8MR46.1, and AtGASA14 is Q9LFR3.1.

## Abbreviations

GASA: Gibberellic acid-stimulated Arabidopsis
GA: Gibberellin
ABA: Abscisic Acid
ET: Ethylene
SA: Salicylic Acid
BR: Brassinolide
CDS: Coding Sequence
Y1H: Yeast One-Hybrid
3-AT: 3-Amino-1,2,4-Triazole
Y2H: Yeast Two-Hybrid
WT: Wild-Type
TR: Transgenic
AMPs: Antimicrobial peptides
SUMO: Small Ubiquitin-like Modifier
