# Supplementary material for: The study on interacting factors and functions of GASA6 in Jatropha curcas L
Source: BMC Plant Biol. 2023 Feb 18;23:99. doi: 10.1186/s12870-023-04067-4 (PMC9938578; doi:10.1186/s12870-023-04067-4)
Supplement: Supplementary file 1 — Additional file 1. [file 12870_2023_4067_MOESM1_ESM.docx]

**
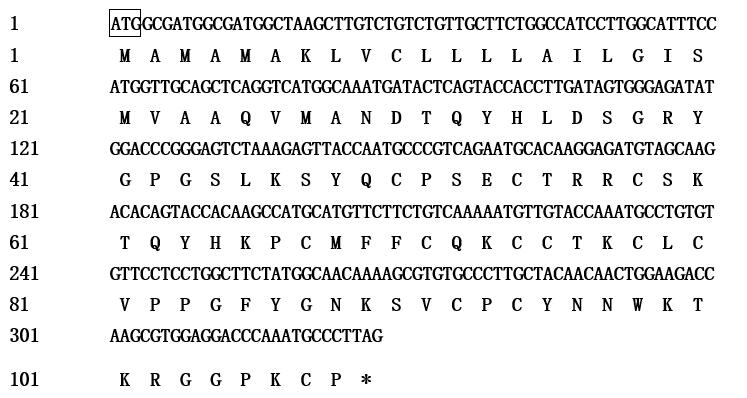

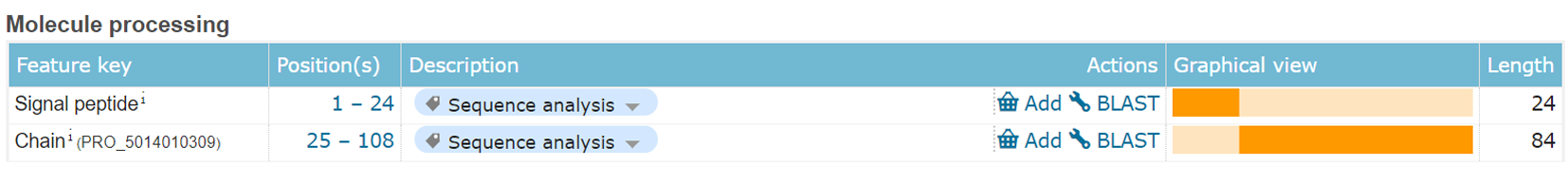
Fig. S1 Amino acid sequence of JcGASA6**

**
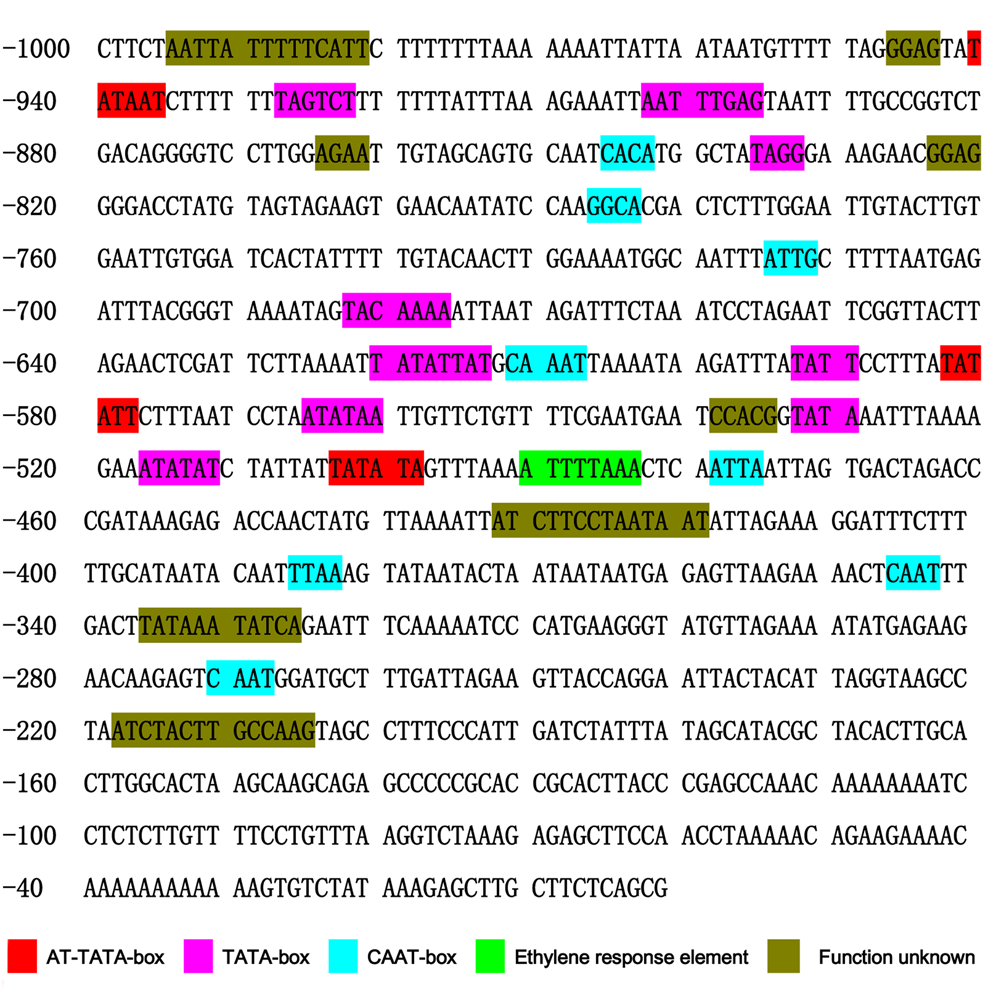
Fig. S2 Analysis of JcGASA6** **signal peptide** **predicted from UniProt.**

**Fig. S3 Promoter sequence characteristics of *JcGASA6*.**

**
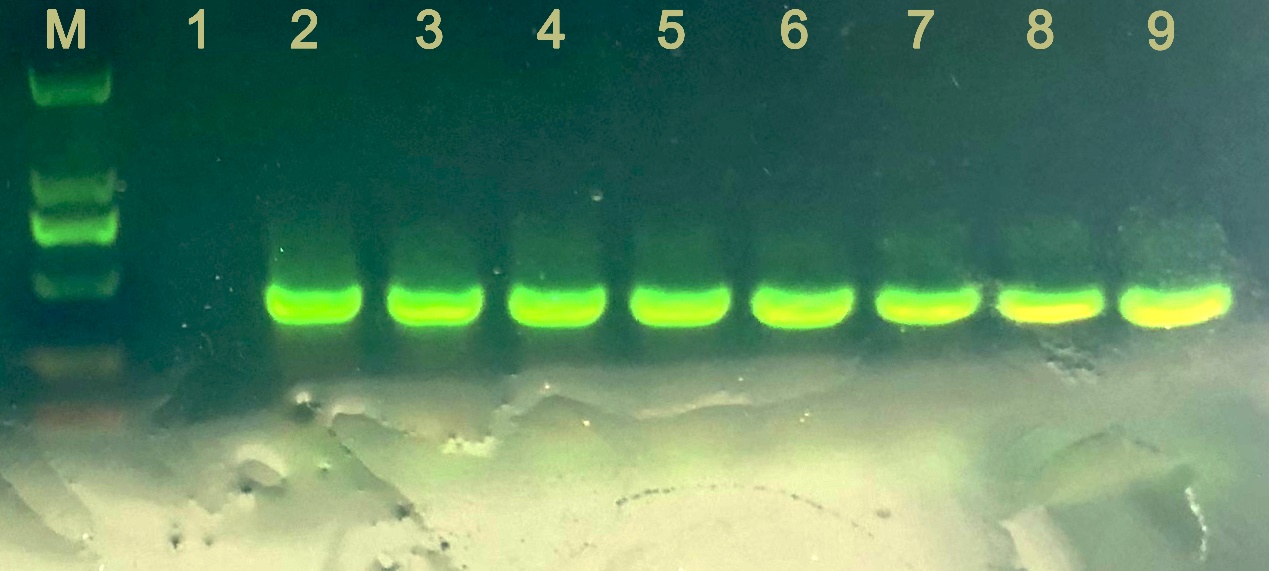

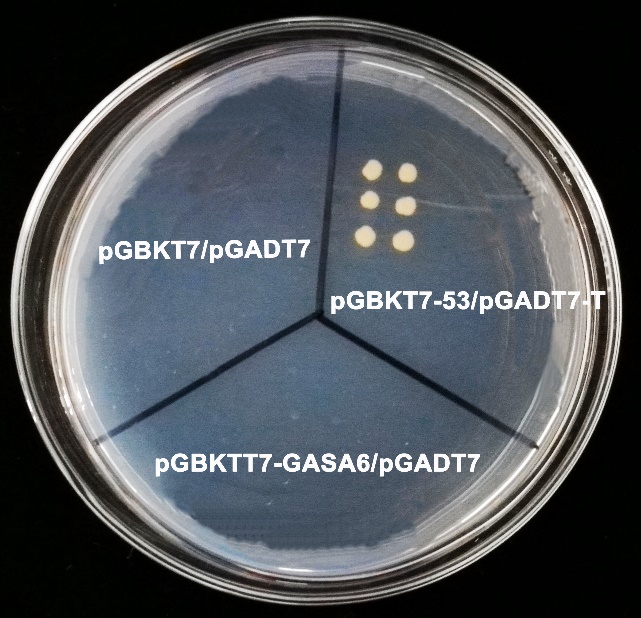
Fig. S4** **Detection of transcriptional self activation of *JcGASA6***

**Fig. S5 The positive** **transgenic tobacco identify by PCR**

M: 2000 marker, 2000 bp, 1000 bp, 750 bp, 500bp, 250 bp, 100 bp. 1: negative transgenic tobacco. From 2 to 9: positive transgenic tobacco.
